# Partial monosomy14q involving *FOXG1* and *NOVA1* in an infant with microcephaly, seizures and severe developmental delay

**DOI:** 10.1186/s13039-016-0269-1

**Published:** 2016-08-02

**Authors:** H. Fryssira, E. Tsoutsou, S. Psoni, S. Amenta, T. Liehr, E. Anastasakis, Ch Skentou, A. Ntouflia, I. Papoulidis, E. Manolakos, N. Chaliasos

**Affiliations:** 1 Medical genetics, School of Medicine, National and Kapodistrian University of Athens, “Aghia Sophia” Children’s Hospital, Athens, Greece; 2 “Mitera” Maternity Hospital, Athens, Greece; 3 “Access to Genome” Clinical Laboratory Genetics, Athens Thessaloniki, Greece; 4 Child Health Department, University Hospital of Ioannina (UHI), Ioannina, Greece; 5 “Mitera kai emvrio” Medical centre, Larisa, Greece; 6 Institute of Human Genetics, Jena University Hospital, Friedrich Schiller University, Jena, Germany; 7 Hellenic Navy Hospital, Deinokratous 70, Athens, 11521 Greece; 8 Department of Medical Genetics, Binaghi Hospital, University of Cagliari, Cagliari, Italy

**Keywords:** FOXG1 syndrome, Rett syndrome, *NOVA1*, Array-CGH, Postnatal microcephaly, Seizures

## Abstract

**Background:**

*FOXG1* gene mutations have been associated with the congenital variant of Rett syndrome (RTT) since the initial description of two patients in 2008. The on-going accumulation of clinical data suggests that the FOXG1-variant of RTT forms a distinguishable phenotype, consisting mainly of postnatal microcephaly, seizures, hypotonia, developmental delay and corpus callosum agenesis.

**Case presentation:**

We report a 6-month-old female infant, born at 38 weeks of gestation after in vitro fertilization, who presented with feeding difficulties, irritability and developmental delay from the first months of life. Microcephaly with bitemporal narrowing, dyspraxia, poor eye contact and strabismus were also noted. At 10 months, the proband exhibited focal seizures and required valproic acid treatment. Array-Comparative Genomic Hybridization revealed a 4.09 Mb deletion in 14q12 region, encompassing the *FOXG1* and *NOVA1* genes. The proband presented similar feature with patients with 14q12 deletions except for dysgenesis of corpus callosum. Disruption of the *NOVA1* gene which promotes the motor neurons apoptosis has not yet been linked to any human phenotypes and it is uncertain if it affects our patient’s phenotype.

**Conclusions:**

Since our patient is the first reported case with deletion of both genes (*FOXG1-NOVA1*), thorough clinical follow up would further delineate the Congenital Rett-Variant phenotypes.

## Background

Since 2008, when mutations in the *FOXG1* gene were initially described in two patients with Rett-like symptoms, more than 90 patients have been reported to have mutations involving the *FOXG1* gene [1–6]. Rett syndrome is typically an X-linked neurodegenerative condition [6] that was initially described by Andreas Rett in 1966. Common clinical features of Rett syndrome include postnatal microcephaly, autism, seizures, breathing abnormalities, growth retardation and gait apraxia [7, 8]. The majority of patients with typical Rett syndrome carry mutations in the gene encoding Methyl-CpG-binding protein 2 (*MECP2*) located at Xq28. Variant forms of Rett syndrome have been revised in 2010 by Neul et al [7]. He classified them in to three main variant categories: 1. Preserved speech, 2. early seizures and, 3. the congenital variant. Mutations in the cyclin-dependent kinase-like 5 (*CDKL5)* gene located in Xp22 are found in most cases of early-onset seizure variants, while the congenital variant of the syndrome has recently been found to be associated with heterozygous mutations or deletions in the forkhead box protein G1 (*FOXG1*) gene, located in the 14q12 chromosomal region [7, 9–11]. In this study, we report a new case of atypical Rett Syndrome with a 14q12 deletion, 4.09 Mb in size, encompassing only two genes, the *FOXG1* and the *NOVA1* and we discuss the role of the *NOVA1* haploinsufficiency.

## Case presentation

Our patient was a 6-month- old female infant and the only child of non-consanguineous parents. She was born by caesarean section at 38 weeks of gestation. Her birth weight was 2.990 gr (25^th^ -50^th^ centile), her length was 49 cm (25^th^ -50^th^ centile) and her head circumference was 33 cm (10–25th centile).

It should be noted that the embryo was the product of in vitro fertilization (IVF) by intracytoplasmic sperm injection (ICSI) due to the husband’s low sperm count. The mother underwent treatment with Follitropin Beta (Puregon), human Chorionic Gonadotropin, hCG (Pregnyl), and Cetrorelix Acetate (Cetrotide).

**Fig. 1:**
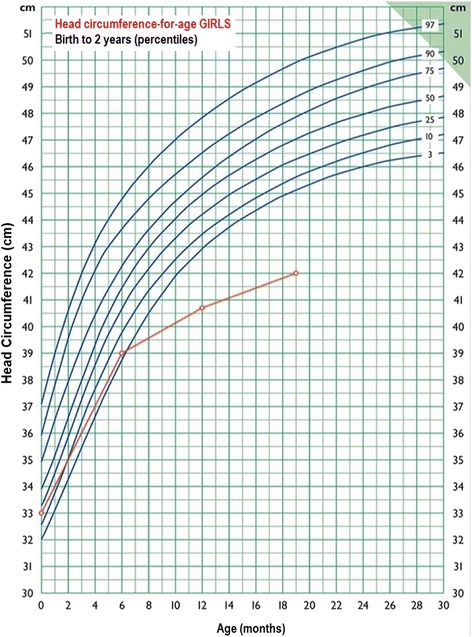
Growth curve ¨Head circumference—for—age GIRLS. First Paediatric department, National and Kapodistrian University of Athens. Professor G.Chroussos. The standard curves are used by the Greek National Health System

The patient as a neonate was hypotonic. She was referred for clinical evaluation at the age of 6 months due to irritability and feeding difficulties. The examinations revealed peripheral hypertonia, psychomotor retardation (limited facial expression—no smile, poor eye contact), borderline microcephaly (head circumference of 39 cm, at 3^rd^ centile) (Fig. 1a), periodical stereotypic movements and minor dysmorphic features such as a small forehead with bitemporal narrowing, strabismus, broad base to nose and long philtrum. Her weight was 6.18 kg (25th centile) and her height was 62 cm (25th centile).

Due to gastroesophageal reflux she was treated with ranitidine.

Haematological, biochemical, metabolic, thyroid, ammonia and blood gasses were all normal. The ultrasounds of the heart and abdomen were also normal.

At 10 months the proband exhibited focal seizures and required valproic acid treatment. Sleep deprived EEG examination revealed slower focal activity for her age and sleep spindles which are indicative signs of focal cerebral dysfunction. Brain magnetic resonance imaging (MRI) showed no serious findings except for a septum pellucidum cyst and brain asymmetry—the left hemisphere was larger than the right one.

At the age of 12 months, the head circumference was 40.7 cm (<< 3^rd^ centile) (Fig. 1a) while the height and weight remained between 10^th^ and 25^th^ centile. Severe neurodevelopmental delay was obvious.

Re-examination at the age of 19- months and 2-years, revealed head circumference of 42 cm (<< 3^rd^ centile) (Fig. 1a), height of 75.5 cm (slightly below the 3^rd^ centile) and weight of 9 Kg (slightly below the 3^rd^ centile). The proband exhibited severe psychomotor retardation as she could neither sit independently nor speak at all.

### Chromosome and array-based analysis

Metaphase chromosomes were obtained from phytohemagglutinin (PHA)-stimulated peripheral blood lymphocytes and high resolution (550–650 bands) thymidine treatment G-banding karyotype analysis was performed, using standard procedures. Twenty metaphase spreads were analysed, and the result was a normal female karyotype (46, XX). The molecular analysis of the *MECP2* gene was normal also.

**Fig. 2:**
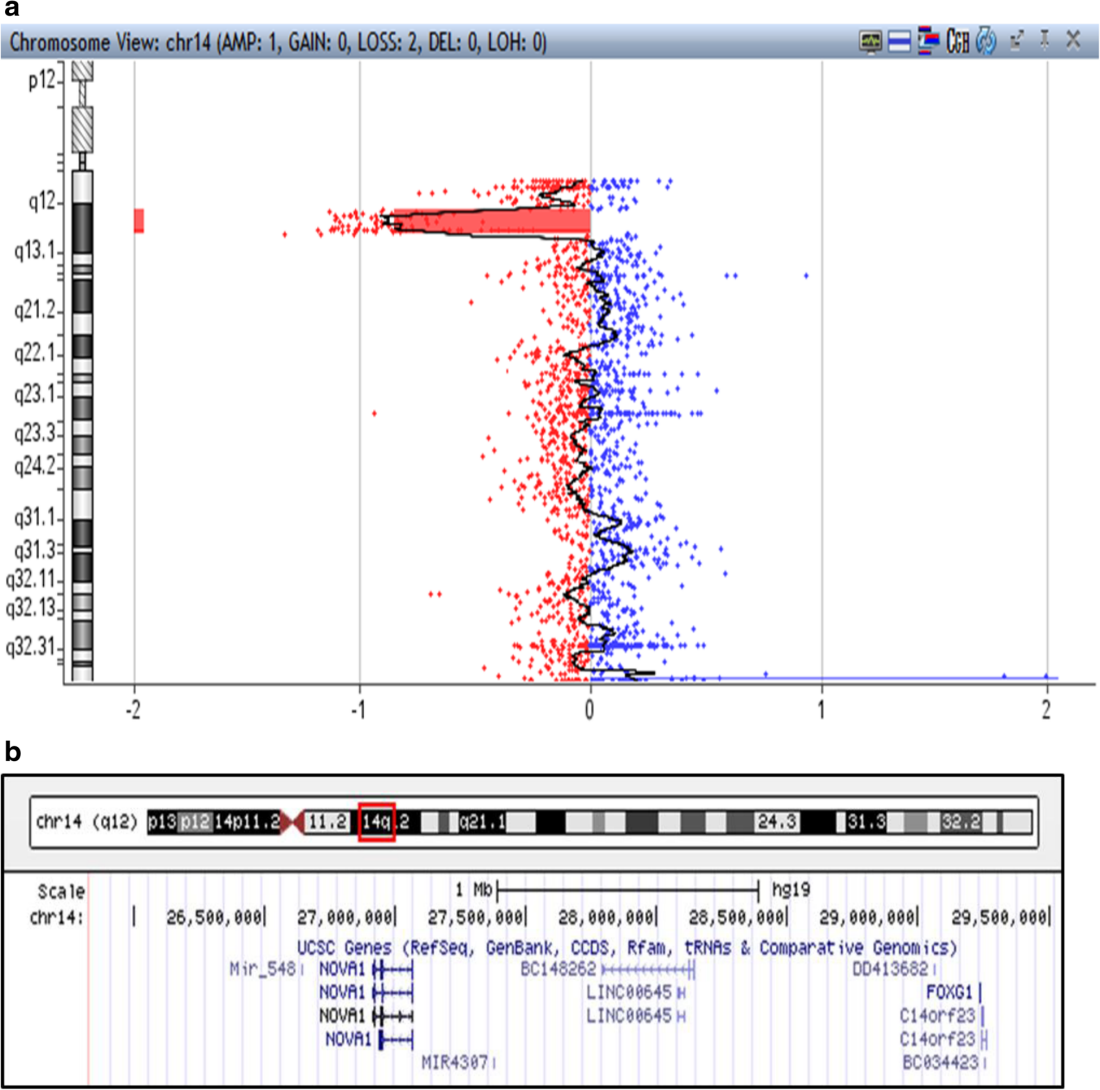
Array-CGH analysis detected a 4.09 Mb loss of the copy numbers in the spanning region 14q12. **b** Chromosome 14, region 25,843,560-29,938,629. Represents the 4,095,070 bp deletion described in our case report. The region includes both *FOXG1* and *NOVA1* genes. Figure adapted from http://genome.ucsc.edu/ Accessed at 18/10/2015

Further investigation with array-Comparative Genomic Hybridization was performed by hybridizing the sample against a male human reference commercial DNA sample (Promega biotech) using an array-CGH platform that includes 60,000 oligonucleotides distributed across the entire genome (Agilent Technologies). The statistical test used as parameter to estimate the number of copies was ADAM-2 (provided by the DNA analytics software, Agilent Techn.) with a window of 0.5 Mb, A = 6. Only those copy number changes that affect at least 5 consecutive probes with identically oriented change were considered as Copy Number Variations (CNV). For the majority of the genome, the average genomic power of resolution of this analysis was 200 kilobases. Array-CGH analysis detected a 4.09 Mb loss in the 14q12 region (Fig. 2a). The deleted segment was mapped at chr14:25,843,560-29,938,629 region. The genomic coordinates are listed according to genomic build GRCh37/hg19, and includes *FOXG1* and *NOVA1* genes (Fig. 2b).

**Fig. 3:**
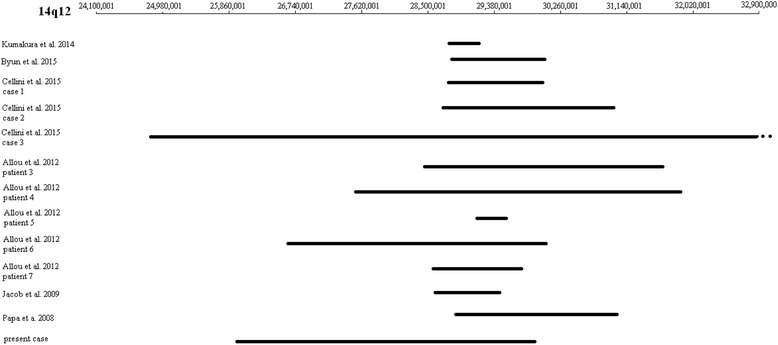
Ideogram of the deletions described in Table 1. The upper thin line indicates the 14q12 region (hg38), and the bold lines indicate the region which is deleted in each case. Cellini et al. 2015 case 3 has a deletion beyond 14q12 and is drawn with a discontinued line

The result of array-CGH was confirmed with FISH. Ten metaphases were analysed from phytohemagglutinin (PHA)-stimulated peripheral blood lymphocytes of the patient with RP11-120I18 in 14q12 and wcp 14 probes. The rearrangement was molecular cytogenetically unbalanced (data not shown), therefore the karyotype is: 46,XX, del[12](q12q12).ish14q12(RP11-120I18-). arr[hg19]14q12(25,843,560-29,938,629)x1. Blood samples from both parents were examined with FISH in order to exclude any chromosome rearrangement like inversion. Ten metaphases were analysed from each subject with RP11-332 N6 in 14q11.2, RP11-120I18 in 14q12 and wcp 14 probes, and no rearrangement was detected (data not shown).

## Discussion

Recently, the *FOXG1* congenital variant of Rett syndrome has been described as a clinically identifiable phenotype, called the “*FOXG1* syndrome” [13]. This is an epileptic-dyskinetic developmental encephalopathy with features of classic Rett syndrome, but earlier onset from the first months of life. The main phenotype comprises postnatal microcephaly, severe developmental delay and lack of speech, hypotonia, dyskinesia and corpus callosum hypoplasia. Other symptoms include strabismus, feeding difficulties, bruxism and seizures [8, 12–16].

In the present study, a novel *FOXG1* and a *NOVA1* deletion is described. According to the diagnostic criteria for typical and atypical Rett syndrome [7], our patient possesses only some of the diagnostic criteria for the atypical Rett syndrome. She has slight facial dysmorphism, feeding difficulties, severe psychomotor retardation, postnatal microcephaly, seizures and focal cerebral dysfunction, strabismus and hypertonia. A deletion of 4.09 Mb on chromosome 14q12 including the *FOXG1* and *NOVA1* genes was identified by array-CGH technique*.* The clinical phenotypes vary between the reported cases that have mutations or deletions involving the *FOXG1* gene and other genes (Table 1, Fig. 3). Our case differs as only two genes, *FOXG1* and *NOVA1*, are involved. These genes were found to be highly expressed in the human brain throughout ontogeny, and double strand breaks in 14q12 region are implicated in the neurodegenerative disease: ataxia telangiectasia [17].

**Table 1:** Clinical presentation of patients with FOXG1 mutations and 14q12 deletions

	*Kumakura* et al*. 2014*	*Byun* et al*. 2015*	*Patients (n = 8) Cellini* et al*. 2015*					*Patients (n = 7) Allou* et al*. 2012*	*Patients (n = 2) Philippe* et al*. 2011*	*Jacob* et al*. 2009*	*Yeung* et al*. 2009*	*Patients (n = 2) Ariani* et al*. 2008*	*Papa* et al*. 2008*	*Present case*
			*1*	*2*	*3*	*4*	*4 cases with point mutations in FOXG1*							
Age (Y,M)	8.0	8.0	9.0	3.6	2.0	10.0	2.6–17.0	N/A	22.0 and 10.0	3.0	9.0	22.0 and 7.0	7.0	0.6
Sex	M	F	F	M	F	F	3 F, 1 M	4 F, 3 M	F	F	F	F	F	F
FOXG1 mutation on 14q12 CNV (Mb)	0.54 Mb Del	2.5 Mb Del	2.5 Mb Del	2.8 Mb Del	9.1 Mb Del	7.3 Mb Dup (de novo)	p.Gln100Serfs*92, p.Q46X, p.Glu154Glyfs*301, p.Gln86Argfs*106 (de novo)	0.4–4.1 Mb	p.Trp308X and p.Tyr400X	2.594 Mb Del	4.45 Mb Dup	p.W255X and p.S323fsX325	3 Mb Del	4,09 Del
Genes involved in mutation or point mutations	*FOXG1, C14orf23 (de novo)*	*FOXG1, C14orf23, PRKD1* (de novo)	*FOXG1, C14orf23, PRKD1*	*FOXG1, C14orf23, PRKD1, SCFD1, G2E3, COCH, STRN3* (de novo)	*STXBP6, NOVA1, FOXG1, C14orf23, PRKD1, SCFD1, G2E3, COCH, STRN3, AP4S1, HECTD1, DTD2, NUBPL, ARHGAP5, AKAP, NPAS3* (de novo)	*FOXG1 and 50 additional genes (de novo)*	*FOXG1*	*Deletions in 5 cases, 3 of them distal to FOXG1 and 2 cases of point mutations involving FOXG1*	*FOXG1*	*FOXG1*, *C14orf23*	*FOXG1, NOVA1, c14orf23, PRKD1, G2E3, SDFD, COCH*	*FOXG1*	*FOXG1, PRKD1, SCFD1, G2E3, COCH, STRN3*	*FOXG1, NOVA1*
Postnatal microcephaly	yes	yes	yes	yes	yes	no	yes	yes	yes	yes	no	yes	yes	yes
Psychomotor retardation	yes	yes	yes	yes	yes	yes	yes	yes	yes	yes	yes	yes	yes	yes
Hypotonia	yes	no	yes	yes	yes	yes	2/4 cases hypotonic	All except 1 case	1/2 cases	yes	yes	yes	yes	Hypertonia
Diskinesia	yes	yes	yes	yes	yes	yes	3/4 cases diskinetic	3/7 cases	yes	yes	yes	yes	yes	yes
Speech	no	no	no	no	no	no	no	no	no	no	no	no	no	no
Hand stereotypies	no	other sterotypies	yes	no	yes	yes	yes	4/7 cases	yes	yes	hand flapping only	yes	yes	yes
Corpus callosum hypogenesis	yes	no	yes	yes	yes	no	3/4 cases with hypoplasia	brain abnormalities in all cases	1/2 cases	no, brachycephaly observed	no	yes	no	no
Seizures	yes	yes	yes	yes	yes	yes	yes	5/7 cases	1/2 cases	yes	yes	yes	yes	yes
Developmental delay	yes	yes	yes	yes	yes	yes	yes	5/7 cases	1/2 cases	yes	yes	yes	yes	yes

*F* female, *M* male, *Y* years, *M* months, *Del* deletion, *Dup* duplication

Different clinical features have also been described in patients with *FOXG1* point mutations. The severity or lack of the symptoms among cases could depend additionally on genetic and/or environmental factors [13]. Jacob F. et al. in 2009 [9] reported a case of a 2.594 Mb deletion involving the *FOXG1* and *C23orf14* (Table 1). As in our case, patient had a normal corpus callosum but showed all the typical characteristics found in patients with *FOXG1* alterations. Table 1 indicates that almost all cases suffer from microcephaly, dyskinesia, lack of speech, seizures and developmental delay. On the other hand, hypotonia and corpus callosum hypoplasia are not apparent in every case. Progressive hypertonia was obvious in our patient and in at least 10 other reported cases with *FOXG1* point mutations [8].

Forkhead BOX G1 *(FOXG1)* gene (OMIM*164874) encodes a developmental transcription factor with repressor activity, important for the development of the ventral telencephalon in the embryonic forebrain and crucial for the regulation of neurogenesis and neurite outgrowth. It is also expressed in neurogenetic regions of the postnatal brain [15]. Postnatal microcephaly is another common feature of FOXG1 syndrome. In cases with a duplication of the *FOXG1* gene, microcephaly is not found (Table 1), indicating that the phenotype is dependent on *FOXG1* dosage. Furthermore, other reported cases with deletions close but not disturbing the *FOXG1* gene have a similar phenotype as patients with ¨FOXG1 syndrome¨, suggesting that a position effect causing altered expression of *FOXG1* gene may be the cause. The protein Kinase D1 (*PRKD1)* gene (OMIM*605435), located close to *FOXG1* gene has been reported to be implicated in transcription and expression of *FOXG1* gene [14].

Neuro-Oncological Ventral Antigen 1 (*NOVA1)* gene (OMIM*602157) encodes a neuron-specific RNA-binding protein that is inhibited by paraneoplastic antibodies [18]. Recently, Storchel H et al., 2015 [19] found that NOVA1 protein converges on Ago proteins and controls miRNA-induced silencing complex (miRISC), possibly resulting on the regulation of neuronal development and synaptic plasticity. Despite the significant role of the *NOVA1* gene, it has not been linked to any human phenotype yet. In the case of our patient with FOXG1 syndrome, we are unable to estimate the impact of the *NOVA1* haploinsufficiency. *NOVA1* haploinsufficiency may act synergistically with the *FOXG1* gene or it may cause no difference in the final clinical outcome. Also, we cannot define whether the IVF by ICSI plays a role in the microdeletion of chromosome 14q12 region. Further research should investigate if ICSI could be associated with haploinsufficiency as was the case in our patient.

In conclusion, we present a female patient with clinical features compatible with the congenital variant of Rett syndrome.

## Conclusion

Since our patient is the only one with a deletion encompassing only *FOXG1* and *NOVA1* genes, in the future the thorough clinical follow-up of her neurological status will help for further delineation of the role of these genes.

## Abbreviations

CGH, comparative genomic hybridization; EEG, electroencephalogram; ICSI, intracytoplasmic sperm injection; IVF, in vitro fertilization; MRI, magnetic resonance imaging; PHA, phytohemagglutinin; RTT, Rett syndrome
